# Supplementary material for: Sorting at embryonic boundaries requires high heterotypic interfacial tension
Source: Nat Commun. 2017 Jul 31;8:157. doi: 10.1038/s41467-017-00146-x (PMC5537356; doi:10.1038/s41467-017-00146-x)
Supplement: Supplementary file 2 — Supplementary Software 1 [file 41467_2017_146_MOESM2_ESM.zip › PottsModel/SrcPottsModel/doc/serialized-form.html]

Serialized Form


JavaScript is disabled on your browser.


Skip navigation links


- Overview
- Package
- Class
- Use
- Tree
- Deprecated
- Index
- Help

- Prev
- Next

- Frames
- No Frames

- All Classes

# Serialized Form

- ## Package gui

  - ### Class gui.ConfigurationInformationPanel extends javax.swing.JPanel implements Serializable

    - ### Serialized Fields

      - #### aText

        ```
        javax.swing.JLabel aText
        ```
      - #### aEngine

        ```
        PottsEngine aEngine
        ```
  - ### Class gui.ConfigurationPanel extends javax.swing.JPanel implements Serializable

    - ### Serialized Fields

      - #### aConstants

        ```
        Constants aConstants
        ```
      - #### vGroup

        ```
        javax.swing.GroupLayout.SequentialGroup vGroup
        ```
      - #### layout

        ```
        javax.swing.GroupLayout layout
        ```
  - ### Class gui.Console extends javax.swing.JPanel implements Serializable

    - ### Serialized Fields

      - #### textArea

        ```
        javax.swing.JTextArea textArea
        ```
      - #### newline

        ```
        java.lang.String newline
        ```
  - ### Class gui.PlotPanel extends javax.swing.JPanel implements Serializable

    serialVersionUID:
    :   -8266389062157718277L

    - ### Serialized Fields

      - #### statNames

        ```
        java.util.List<E> statNames
        ```

        The number of subplots.
      - #### datasets

        ```
        java.util.HashMap<K,V> datasets
        ```

        The datasets.
      - #### lastValue

        ```
        double[] lastValue
        ```

        The most recent value added to series 1.
  - ### Class gui.PottsCanvas extends javax.swing.JPanel implements Serializable

    - ### Serialized Fields

      - #### aIsInitialized

        ```
        boolean aIsInitialized
        ```
      - #### aConstants

        ```
        Constants aConstants
        ```
      - #### aLattice

        ```
        Lattice aLattice
        ```
      - #### aScreenWidth

        ```
        int aScreenWidth
        ```
      - #### aScreenHeight

        ```
        int aScreenHeight
        ```
      - #### aPottsWidth

        ```
        int aPottsWidth
        ```
      - #### aPottsHeight

        ```
        int aPottsHeight
        ```
      - #### aPixelSize

        ```
        int aPixelSize
        ```
      - #### aPixels

        ```
        PixelDisplay[][] aPixels
        ```
      - #### aSnapshot

        ```
        java.awt.image.BufferedImage aSnapshot
        ```
      - #### spcSupport

        ```
        javax.swing.event.SwingPropertyChangeSupport spcSupport
        ```
      - #### aFrame

        ```
        PottsFrame aFrame
        ```
  - ### Class gui.PottsFrame extends javax.swing.JFrame implements Serializable

    - ### Serialized Fields

      - #### aCanvas

        ```
        PottsCanvas aCanvas
        ```
      - #### aToolbar

        ```
        PottsToolbar aToolbar
        ```
      - #### aStatusBar

        ```
        StatusBar aStatusBar
        ```
      - #### aConsole

        ```
        Console aConsole
        ```
      - #### aEngine

        ```
        PottsEngine aEngine
        ```
      - #### aSidePane

        ```
        javax.swing.JTabbedPane aSidePane
        ```
      - #### aInfo

        ```
        ConfigurationInformationPanel aInfo
        ```
      - #### cStatsPanelIndex

        ```
        int cStatsPanelIndex
        ```
      - #### aPaused

        ```
        java.util.concurrent.atomic.AtomicBoolean aPaused
        ```
      - #### aSwapAndPause

        ```
        java.util.concurrent.atomic.AtomicBoolean aSwapAndPause
        ```
      - #### aMCSAndPause

        ```
        java.util.concurrent.atomic.AtomicBoolean aMCSAndPause
        ```
      - #### aAutomaticMode

        ```
        java.util.concurrent.atomic.AtomicBoolean aAutomaticMode
        ```
  - ### Class gui.PottsToolbar extends javax.swing.JPanel implements Serializable

    serialVersionUID:
    :   1789315475550161831L

    - ### Serialized Fields

      - #### aFrame

        ```
        PottsFrame aFrame
        ```
      - #### textArea

        ```
        javax.swing.JTextArea textArea
        ```
      - #### newline

        ```
        java.lang.String newline
        ```
      - #### swapButton

        ```
        javax.swing.JButton swapButton
        ```
      - #### mcsButton

        ```
        javax.swing.JButton mcsButton
        ```
      - #### autoButton

        ```
        javax.swing.JButton autoButton
        ```
  - ### Class gui.StatusBar extends javax.swing.JPanel implements Serializable

    serialVersionUID:
    :   6172502704925664980L

    - ### Serialized Fields

      - #### textArea

        ```
        javax.swing.JTextArea textArea
        ```
      - #### newline

        ```
        java.lang.String newline
        ```
      - #### aMCSLabel

        ```
        javax.swing.JLabel aMCSLabel
        ```

Skip navigation links


- Overview
- Package
- Class
- Use
- Tree
- Deprecated
- Index
- Help

- Prev
- Next

- Frames
- No Frames

- All Classes
